# Efficacy and safety of telithromycin 800 mg once daily for 7 days in community-acquired pneumonia: an open-label, multicenter study

**DOI:** 10.1186/1471-2334-5-43

**Published:** 2005-05-31

**Authors:** Charles M Fogarty, Tushar C Patel, Lala M Dunbar, Bruno P Leroy

**Affiliations:** 1 Spartanburg Medical Research, Spartanburg, South Carolina, USA; 2 Fall River Walk-in Clinic, Fall River, Massachusetts, USA; 3 Department of Medicine/Emergency Medicine, Louisiana State University Medical Center, New Orleans, Louisiana, USA; 4 Aventis Pharmaceuticals, Bridgewater, New Jersey, USA

## Abstract

**Background:**

Community-acquired pneumonia (CAP) remains a major cause of morbidity and mortality throughout the world. Telithromycin (a new ketolide) has shown good *in vitro *activity against the key causative pathogens of CAP, including *S pneumoniae *resistant to penicillin and/or macrolides.

**Methods:**

The efficacy and safety of telithromycin 800 mg orally once daily for 7 days in the treatment of CAP were assessed in an open-label, multicenter study of 442 adults.

**Results:**

Of 149 microbiologically evaluable patients, 57 (9 bacteremic) had *Streptococcus pneumoniae*. Of the 57 *S pneumoniae *pathogens isolated in these patients, 9 (2 bacteremic) were penicillin- or erythromycin-resistant; all 57 were susceptible to telithromycin and were eradicated. Other pathogens and their eradication rates were: *Haemophilus influenzae *(96%), *Moraxella catarrhalis *(100%), *Staphylococcus aureus *(80%), and *Legionella *spp. (100%). The overall bacteriologic eradication rate was 91.9%. Of the 357 clinically evaluable patients, clinical cure was achieved in 332 (93%). In the 430 patients evaluable for safety, the most common drug-related adverse events were diarrhea (8.1%) and nausea (5.8%).

**Conclusion:**

Telithromycin 800 mg once daily for 7 days is an effective and well-tolerated oral monotherapy and offers a new treatment option for CAP patients, including those with resistant *S pneumoniae*.

## Background

Community-acquired pneumonia (CAP) remains a major cause of morbidity and mortality throughout the world. In the USA alone, an estimated 3–4 million cases of CAP account for approximately 10 million physician visits, 500,000 hospitalizations, and 45,000 deaths each year [1,2]. Common bacterial pathogens in CAP include *Streptococcus pneumoniae*, *Haemophilus influenzae*, *Moraxella catarrhalis*, *Mycoplasma pneumoniae*, *Chlamydia pneumoniae*, and *Legionella pneumophila*. Ideally, antibiotic therapy for CAP should be based on etiologic diagnosis. The Infectious Diseases Society of America (IDSA) recommends microbiologic testing, including sputum and blood cultures, in hospitalized CAP patients, and encourages sputum culture and Gram staining in CAP outpatients [2]. Practically, however, microbiologic test results are not usually immediately available and a clinician needs to initiate treatment that will cover the most likely pathogens within a few hours of a patient's initial presentation. Complicating the selection of effective empiric therapy is the possibility that a patient may ultimately prove to have a resistant pathogen, an atypical/intracellular pathogen, or both. The increasing prevalence of drug resistance among the typical respiratory tract pathogens is now an important consideration in selecting an antibacterial agent for CAP [2]. High resistance to penicillin (a previously effective therapy for pneumococcal pneumonia) is now > 15% in some areas, and an increasing proportion of *S pneumoniae *strains are now macrolide-resistant [3,4].

Recognizing the increasing resistance of typical pathogens and increasing incidence of atypical/intracellular pathogens, the American Thoracic Society (ATS) has listed a new class of agents – the ketolides, which belong to the macrolide-lincosamide-streptogramin_B _(MLS_B_) family – as a potential option for oral therapy in CAP [5]. The ketolide telithromycin has shown good *in vitro *activity against *S pneumoniae *resistant to penicillin and/or macrolides [6], and potent activity against both typical and atypical/intracellular respiratory tract pathogens [6-17]. *In vitro *studies have shown that telithromycin has a low potential to select for resistant strains and does not induce MLS_B _resistance [18-20]. A once-daily dose of telithromycin 800 mg yields drug concentrations in plasma and bronchopulmonary tissues and fluids in excess of the minimum inhibitory concentration (MIC) of most common respiratory pathogens for 12–24 hours after dosing [21,22]. Concentrations of telithromycin in alveolar macrophages, epithelial lining fluid, and bronchial mucosa 8 hours after a single 800 mg dose exceed those in plasma by factors of 180, 6.5, and 2.2, respectively [21].

The principal objective of the present study was to evaluate the bacteriologic and clinical efficacy and safety of a 7-day regimen of telithromycin 800 mg once daily in patients with CAP, with a focus on infections due to penicillin- and/or erythromycin-resistant *S pneumoniae*.

## Methods

### Patient sample

Both in-patients and outpatients ≥ 13 years of age were considered for inclusion in the study. The diagnosis of CAP was based on a new chest x-ray infiltrate and the presence of ≥ 2 of the following: cough; production of purulent sputum; auscultatory findings on pulmonary examination; dyspnea; fever; elevated white blood cell count; or a positive Gram stain in a sputum sample. The decision to admit was based on the clinical judgment of the patient's physician. Women of childbearing potential were required to have a negative pregnancy test and use an accepted method of contraception throughout the study.

Exclusion criteria included: need for parenteral antibacterial therapy; antibacterial therapy for > 24 hours in the previous 7 days; use of azithromycin, ceftriaxone, or dirithromycin within 7 days prior to enrollment; empyema or lung abscess; pulmonary disease requiring a specific treatment that would make the interpretation of the results difficult; suspected nonbacterial pathogen; pathogen resistant to the study medication; known long QTc syndrome; sick sinus syndrome; severe hypokalemia; hypersensitivity to macrolides; terminal illness or immunocompromised status; concomitant medications known to have a potential interaction with telithromycin; and impaired hepatic or renal function, or clinically relevant cardiovascular, neurologic, or endocrine disease.

### Study design

Patients were enrolled between January and September 2000 at 37 centers in the USA, 10 centers in South America, 6 centers in South Africa, and 5 centers in Canada. The trial was conducted in accordance with good clinical practice, and the study protocol and other appropriate study-related documents were reviewed and approved by the respective independent ethics committees/institutional review boards in the participating countries. All patients provided written informed consent.

The study groups evaluated comprised:

• Safety population: All patients who received ≥ 1 dose of telithromycin and had ≥ 1 safety assessment after entry into the study

• Modified intent to treat (mITT) population: All patients with a confirmed diagnosis of CAP who received ≥ 1 telithromycin tablet

• Per-protocol clinically evaluable (PPc) population: All mITT patients who completed the study with no major protocol violations

• Per-protocol bacteriologically evaluable (PPb) population: All clinically evaluable patients who also had a bacterial pathogen identified as a causative agent

• Bacteriologic modified intent to treat (bmITT) population: All mITT patients with a bacteriologic sample obtained at pretherapy/entry containing ≥ 1 causative pathogen.

All patients meeting the study inclusion criteria were assigned to 7 days of open-label treatment with telithromycin 800 mg, administered once daily in the morning as two 400 mg tablets. Compliance with treatment was measured by tablet count at the on-therapy visit (Days 3–5) and at or before the post-therapy/test of cure (TOC) visit.

### Study endpoints

Bacteriologic outcome in the PPb patients at the post-therapy/TOC visit was the primary efficacy variable. The secondary efficacy variables in the study were bacteriologic outcome in the mITT patients who had ≥ 1 causative pathogen identified at entry (bmITT), and clinical outcome at the post-therapy/TOC visit in the PPc and mITT patients. Special attention was given to infections due to *S pneumoniae *resistant to penicillin and/or erythromycin.

### Bacteriologic evaluation

Sputum samples were obtained for Gram stain, culture, and susceptibility analyses at the 4 study visits: pretherapy/entry (Day 1), on-therapy (Days 3–5), end-of-therapy (Days 8–10), and post-therapy/TOC (Days 17–24), or at early withdrawal if applicable. Blood samples for culture were obtained at pretherapy/entry and at subsequent assessments if cultures were previously positive, a febrile state persisted, or no clinical improvement had occurred after 48 hours of treatment. Susceptibility of pathogens to telithromycin was tested using disk diffusion and dilution methods. Susceptibility to macrolides (i.e. erythromycin) was tested using disk diffusion to establish a minimal resistance profile to these agents for the pathogens isolated. Oxacillin disks were used to screen for penicillin resistance among *S pneumoniae *isolates, and the nitrocephin test was used to detect β-lactamase production in *H influenzae *and *M catarrhalis*.

Tests for detection of atypical/intracellular pathogens included serologic testing of blood samples and/or polymerase chain reaction (PCR) analysis of oropharyngeal swab samples or sputum samples for *C pneumoniae *and *M pneumoniae*, and urine and blood samples for *L pneumophila*. Diagnostic criteria for atypical/intracellular pathogens included negative cultures for common pathogens and the following: for *L  pneumophila*, a 4-fold increase in paired serum immunoglobulin (Ig) G or IgM titers, or a positive urinary antigen for *L pneumophila *serogroup I; for *C pneumoniae*, a single IgM titer ≥ 1:32 in combination with a positive PCR, or a 4-fold increase in paired serum IgG/IgM titers; for *M pneumoniae*, a 4-fold increase in paired serum IgG or a single IgM titer ≥ 1:16 with a positive PCR test. The tests used to confirm the presence of *C pneumoniae *were based upon approved/agreed FDA procedures, including DNA gene probe of the sputum, acute and convalescent serology for antibodies from the blood, and microimmunofluorescent antigen testing of sputum. *M pneumoniae *tests were based upon acute and convalescent serology and DNA gene probe. Convalescent serology was not performed on patients initially positive for common pathogens.

Bacteriologic outcome was based on eradication, presumed eradication, or persistence of any pathogen isolated in microbiologic cultures at the pretherapy/entry visit and considered by the investigator to be causative of CAP. Assessments also included analyses for the presence of any new pathogen isolated during or after treatment that was not detected at the pretherapy/entry visit. Bacteriologic outcome was categorized as satisfactory if the causative pathogen was absent in on-therapy or post-therapy cultures (eradication), or if no follow-up culture was available due to clinical improvement (presumed eradication). Bacteriologic outcome was considered unsatisfactory if the causative pathogen was still present (persistence), additional antibacterial therapy was indicated (presumed persistence), a new pathogen emerged during therapy or within 3 days after treatment was completed (superinfection), eradication of the causative organism was followed by replacement with a new species or serotype of the same organism (reinfection), or the causative organism reappeared following eradication (recurrence).

Indeterminate outcomes included cases in which the patient was withdrawn from the study before follow-up cultures were obtained, the microbiologic data were incomplete, concurrent antibacterial treatment was provided for reasons not associated with CAP or respiratory tract infections (RTIs), or death occurred that was not due to CAP or related complications. If ≥ 1 causative pathogen was isolated from the pretreatment culture and the bacteriologic outcome was not the same for all pathogens, the bacteriologic outcome was classified as unsatisfactory.

### Clinical evaluation

Patients were evaluated clinically at the four study visits. At entry (Day 1), patients were screened and evaluated for infection-related signs and symptoms, with assessment of vital signs, physical examination, chest x-ray, blood for hematology and chemistry, and a 12-lead electrocardiogram. Similarly, at the on-therapy, end-of-therapy, and post-therapy/TOC visits, patients were assessed for infection-related signs and symptoms, with measurement of vital signs, physical examination and evaluation of overall clinical status, and blood for hematology and chemistry. An electrocardiogram recording was repeated at all visits if the pretherapy/entry QTc was ≥ 500 ms or if the subject was taking a concomitant medication that affected cardiac conduction. A repeat chest x-ray was obtained at the post-therapy/TOC visit.

Evaluation of clinical outcome was based on the investigator's assessment of pneumonia-related signs and symptoms and a chest x-ray at the post-therapy/TOC visit in comparison with the pretherapy/entry visit. Possible clinical outcomes were "cure" (returned to preinfection state or postinfectious stigmata indicative of a normal course of clearance of the infectious process and not requiring further antibiotic treatment), "failure" (residual symptoms or complications of CAP requiring further antibiotic treatment), and "indeterminate" (lost to follow-up or discontinuation not related to study drug).

### Safety evaluation

Adverse events (AEs) were spontaneously reported by the patients and/or observed by the investigator from the time of entry to 14 days after the final dose of telithromycin. These events included any sign, symptom, syndrome, or illness that appeared or worsened and that might impair patient well-being. Investigators assessed causality of AEs as being either possibly related or not related to the study drug. Laboratory safety data analyses (i.e. hematology, blood chemistry, urinalysis) were performed according to standard laboratory procedures by Covance Central Laboratory Services, Indianapolis, Indiana.

### Statistical analysis

Baseline and outcome data for continuous variables were calculated using summary statistics, and for categoric variables using frequency tabulation and calculated percentages. Two-sided 95% confidence intervals (CIs) were calculated for both bacteriologic outcomes and clinical cure rates. AEs were tabulated, and vital signs and QTc intervals were calculated with summary statistics. Post-therapy/TOC clinical laboratory values were compared with baseline using descriptive statistics, and potentially important laboratory values were compared with predefined levels outside the extended normal range for the respective parameter.

## Results

A total of 432 in-patients and outpatients ≥ 13 years of age were enrolled at entry and received open-label treatment with telithromycin; 14 were excluded from the mITT population due to chest x-ray findings that were negative for pneumonia, resulting in an mITT population of 418 patients. Demographic and clinical characteristics of the mITT population are summarized in Table 1. Bacteria other than *S pneumoniae *identified in bacteremic patients included *Streptococcus viridans *and *Staphylococcus haemolyticus*. During the 7-day treatment period, 395(94.5%) patients in the mITT population were 100% compliant.

**Table 1:** Demographic and clinical characteristics for the modified intent to treat population.

**Characteristic**	**n (%)**
**Total treated**	418
**Gender**	241 (57.7)
Male	177 (42.3)
Female	
**Age, years**	45.0 [13–92]
Median range	
< 65	362 (86.6)
≥ 65	56 (13.4)
**BMI**	417
Mean ± SD	26.9 ± 6.9
**Smoking status**	
Smoker	160 (38.3)
Ex-smoker	79 (18.9)
Nonsmoker	179 (42.8)
**Chest x-ray findings**	
Unilateral	304 (73.6)
Bilateral	109 (26.4)
Single lobe*	351 (85.0)
Multiple lobe	54 (13.1)
Pleural effusion	15 (3.6)
**Bacteremia**	15 (3.6)
*S pneumoniae *bacteremia	9 (2.2)
**Investigator assessment of current episode**	
Mild	93 (22.2)
Moderate	279 (66.7)
Severe	46 (11.0)
**PSI**	
Class I	222 (53.1)
Class II	144 (34.4)
Class III	39 (9.3)
Class IV	12 (2.9)
Class V	1 (0.2)

BMI = body mass index; PSI = pneumonia severity index; SD = standard deviation.

*"Single lobe" could apply to either unilateral or bilateral lung involvement (i.e. 1 lobe per right and/or left lung).

Causative pathogens identified at pretherapy/entry in the bmITT (n = 255) and PPb populations (n = 149) are shown in Table 2. Of these, 187/337 (55.5%) isolates in the bmITT population and 119/204 (58.3%) isolates in the bacteriologically evaluable group represented three of the bacteria most commonly implicated in CAP: *S pneumoniae*, *H influenzae*, and *M catarrhalis*. Others included *Klebsiella pneumoniae *and *Staphylococcus aureus*. A typical/intracellular pathogens were detected in a small number of patients: *C pneumoniae*, *M pneumoniae*, and *L pneumophila *in 3, 2, and 4 patients, respectively.

**Table 2:** Causative pathogens identified at pretherapy/entry in the per-protocol bacteriologically evaluable (PPb) and bacteriologic modified intent to treat (bmITT) populations.

**Pathogen**	**Number of Isolates in PPb Patients**	**Number of Isolates in bmITT Patients**
**Total**	204	337
*S pneumoniae*	57	76
*H influenzae*	49	88
*H parainfluenzae*	46	81
*M catarrhalis*	13	23
*K pneumoniae*	3	9
*S aureus*	15	23
Other*	21	37

*Includes: *Haemophilus haemolyticus *(7); *Haemophilus parahaemolyticus *(7); *Enterobacter cloacae *(4); *Pseudomonas aeruginosa *(4); *Acinetobacter baumannii *(1 bmITT only); *Citrobacter freundii *(1 bmITT only); *Enterobacter aerogenes *(1); *Enterobacter agglomerans *(1 bmITT only); *Enterobacter amnigenus *(1 bmITT only); *Enterobacter *not otherwise specified (NOS) (1 bmITT only); *Neisseria meningitides *(1); *Proteus mirabilis *(1); *Proteus NOS *(1 bmITT only); *Streptococcus*, viridansgroup(5); *Staphylococcus haemolyticus *(1).

### Bacteriologic outcome

Satisfactory bacteriologic outcome was achieved in 137/149 (91.9%, 95% CI: 87.5–96.3) patients in the bacteriologically evaluable subgroup and in 215/255 (84.3%, 95% CI: 79.8–88.8) patients in the bmITT subgroup (Table 3). Results in the bmITT population (a secondary efficacy variable) thus support the positive outcome achieved in the bacteriologically evaluable patients. Of the 12 subjects with an unsatisfactory bacteriologic outcome, 6 had pathogens considered persistent (*H influenzae*, *S aureus*, and *Pseudomonas aeruginosa*) and 6 had pathogens classified as presumed persistent (*S aureus*, *Enterobacter cloacae*, and *Enterobacter aerogenes*). Clinical cure was noted in 2 patients with persistent *H influenzae *and 2 with persistent *P aeruginosa*. Eight patients with persistent and presumed persistent bacteria were considered clinical failures.

**Table 3:** Bacteriologic outcomes at the post-therapy/test of cure evaluation for the bacteriologically evaluable and bacteriologic modified intent to treat (bmITT) populations.

	**Bacteriologically Evaluable Population**		**bmITT Population**	
		
**Assessment**	**n (%)**	95% CI*	**n (%)**	95% CI*
n	149		255	
Satisfactory^†^	137 (91.9)	87.5; 96.3	215 (84.3)	79.8; 88.8
Unsatisfactory^‡^	12 (8.1)		40 (15.7)	
Indeterminate	-		16 (6.3)	

*Two-sided 95% confidence interval (CI).

^†^Includes eradication, presumed eradication, and colonization.

^‡^Includes reinfection, superinfection, recurrence, presumed persistence, and persistence.

**Figure 1:**
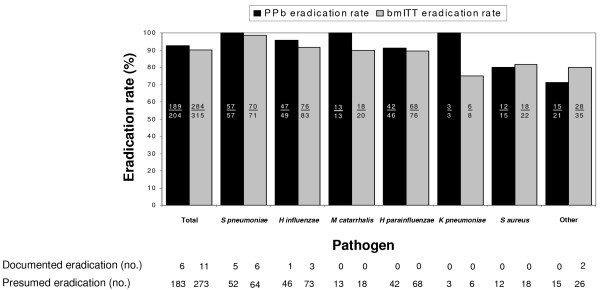
Bacteriologic eradication rates by causative pathogen in the bacteriologically evaluable (PPb) and the bacteriologic modified intent to treat (bmITT) populations at the post-therapy/test of cure (TOC) visit.

Eradication rates, including documented and presumed eradication, at post-therapy/TOC for the bacteriologically evaluable and bmITT populations are depicted in Figure 1. Of 204 pathogens identified in bacteriologically evaluable patients at entry or at subsequent study visits, 189 (92.6%) were eradicated. The eradication rate in the bmITT population was 284/315(90.2%). It is notable that all 57 (100%) of the *S pneumoniae *isolates in the bacteriologically evaluable patients and 70/71 (98.6%) in the bmITT population were eradicated by telithromycin. Eradication rates for *H influenzae *and *M catarrhalis *were also high (95.9% and 100%, respectively) in telithromycin bacteriologically evaluable patients.

*In vitro *susceptibility testing of causative pathogens revealed that 70/71 *S pneumoniae *isolates were susceptible to telithromycin at study entry in the bmITT population, including those exhibiting resistance to other selected antibiotics. One isolate had intermediate susceptibility to telithromycin (MIC 2.00 μg/mL). All *M catarrhalis *and *S aureus *strains isolated in the bacteriologically evaluable and bmITT populations, and 91.8% and 90.9% of *H influenzae *strains isolated in the bacteriologically evaluable and bmITT populations, respectively, were also susceptible to telithromycin (MIC ≤ 1 μg/mL). It is notable that a discrepancy was observed between bacteriologic eradication rates and susceptibility to telithromycin for *S aureus *and *H influenzae*: eradication rates were 80% and 96%, respectively, and telithromycin susceptibility was 100% and 90%, respectively, for the two pathogens.

Nine subjects in the per-protocol bacteriologically evaluable population exhibited *S pneumoniae *strains that were resistant to penicillin or erythromycin. All of these patients (including 2 with bacteremia) had bacteriologic outcomes of eradication or presumed eradication and clinical outcomes of cure (Table 4).

**Table 4:** Bacteriologic and clinical outcome in per-protocol bacteriologically evaluable patients with *S pneumoniae *isolates resistant to penicillin G (Pen G) and/or erythromycin A (Ery A).

		MIC Susceptibility* (μg/mL)				
				
**Subject No.**	**Isolate (Source)**	**TEL**	**Pen G**	**Ery A**	**Bacteriologic Outcome^†^**	**Clinical Outcome**
**Subjects with single-pathogen *S pneumoniae *isolate**						
1	*S pneumoniae *(sputum)	0.030 (S)	0.030 (S)	b>8.000 (R)	Eradication	Cure
2	*S pneumoniae *(sputum)	0.030 (S)	2.000 (R)	0.250 (S)	Presumed eradication	Cure
3	*S pneumoniae *(sputum)	0.500 (S)	2.000 (R)	8.000 (R)	Presumed eradication	Cure
***Subjects with multiple-pathogen S pneumoniae *isolate**						
4	*S pneumoniae *(sputum)	0.060 (S)	2.000 (R)	4.000 (R)	Presumed eradication	Cure
	*M catarrhalis*	0.120 (S)	ND	0.120	Presumed eradication	Cure
5	*S pneumoniae *(sputum)	0.250 (S)	2.000 (R)	8.000 (R)	Presumed eradication	Cure
6	*S pneumoniae *(sputum)	0.030 (S)	2.000 (R)	0.060 (S)	Presumed eradication	Cure
	*H influenzae*	0.002 (S)	ND	1.000	Presumed eradication	Cure
	*M catarrhalis*	0.120 (S)	ND	0.250	Presumed eradication	Cure
7	*S pneumoniae *(sputum)	0.500 (S)	2.000 (R)	8.000 (R)	Presumed eradication	Cure
	*H influenzae*	2.000 (S)	ND	4.000	Presumed eradication	Cure
8	*S pneumoniae *(blood)	1.000 (S)	0.250 (I)	8.000 (R)	Presumed eradication	Cure
	*H influenzae*	1.000 (S)	ND	4.000	Presumed eradication	Cure
9	*S pneumoniae *(blood)	1.000 (S)	0.500 (I)	8.000 (R)	Presumed eradication	Cure
	*H influenzae*	8.000 (R)	ND	4.000	Presumed eradication	Cure
	*S aureus*	0.120 (S)	ND	0.500	Presumed eradication	Cure

MIC = minimum inhibitory concentration; ND = no data; TEL = telithromycin.

*Susceptibility: S = sensitive; I = intermediate; R = resistant.

^†^Bacteriologic outcome.

In the bmITT population subgroup, penicillin resistance (MIC ≥ 2.0 μg/mL), penicillin intermediate resistance (MIC = 0.12–1.0 μg/mL), and erythromycin (macrolide) resistance (MIC ≥ 1.0 μg/mL) were noted in 8/71 (11.3%), 4/71 (5.6%), and 9/71 (12.7%) *S pneumoniae *strains, respectively. Of those strains showing resistance to penicillin, 5/8 (62.5%) were also resistant to the macrolides and all 8 (100%) were resistant to trimethoprim/sulfamethoxazole and cefuroxime axetil. Seven of the macrolide-resistant strains (77.8%) were also resistant to trimethoprim/sulfamethoxazole, and 5 (55.6%) were resistant to cefuroxime axetil based on approved National Committee for Clinical Laboratory Standards susceptibility testing breakpoints.

### Clinical outcome

Clinical cure with telithromycin in the PPc population at the post-therapy/TOC visit was achieved in 332/357 (93.0%) patients overall (Table 5). Clinical cure in the mITT population at the post-therapy/TOC visit was 357/418 (85.4%), reinforcing the clinical outcome achieved by telithromycin in the clinically evaluable patients.

**Table 5:** Clinical outcomes for patients according to demographic characteristics of interest (clinically evaluable population at the post-therapy/test of cure visit)

	**Clinical Outcome**	
	
**Subgroup**	**n**	**Clinical cure, n (%)**
**All patients**	357	332 (93.0)
**Bacteremia**	14	14 (100)
***S pneumoniae *bacteremia**	9	9 (100)
**Age ≥ 65 years**	47	45 (95.7)
**Chest x-ray findings**		
Unilateral	256	236 (92.2)
Bilateral	97	92 (94.8)
Single lobe*	303	282 (93.1)
Multiple lobes	42	38 (90.5)
**Fever >38°C (oral or equivalent)**	123	117 (95.1)
**PSI**		
Class I	187	176 (94.1)
Class II	126	115 (91.3)
Class III	35	32 (91.4)
Class IV	9	9 (100)

PSI = pneumonia severity index.

*"Single lobe" could apply to either unilateral or bilateral lung involvement (i.e. 1 lobe per right and/or left lung).

No patient with a satisfactory bacteriologic outcome had a clinical failure. Clinical cure was achieved for 100% of patients infected with *S pneumoniae*, *H influenzae*, and *M catarrhalis*. Telithromycin also had excellent activity in clinically evaluable patients infected with atypical/intracellular pathogens. Clinical cure was achieved in 100% of patients infected with *C pneumoniae *(n = 3), *M pneumoniae *(n = 2), and *L pneumophila *(n = 4).

Telithromycin was highly effective in patients with risk factors for increased morbidity. In clinically evaluable patients with bacteremia (n = 14), in addition to those with *S pneumoniae *bacteremia (n = 9), telithromycin treatment yielded a clinical cure rate of 100%. In clinically evaluable patients ≥ 65 years of age (n = 47), the clinical outcome was 95.7% (45/47 cured). Patients with multiple lobe involvement had a clinical cure rate of 90.5% (38/42 cured) and those with pneumonia severity index (PSI) scores of III (n = 35) and IV (n = 9) had clinical cure rates of 91.4% and 100%, respectively.

### Safety evaluation

Of 430 patients who received ≥ 1 dose of study medication and who were evaluated for safety, 154 (35.8%) experienced ≥ 1 treatment-emergent adverse event (TEAE) and 87 (20.2%) experienced AEs considered by the investigator to be treatment-related. (All AEs classified as treatment-emergent and possibly treatment-emergent were considered to be treatment-emergent.) Most TEAEs, regardless of causality, were considered by the investigators to be mild or moderate in severity. The two most commonly reported TEAEs were diarrhea in 35 patients (8.1%) and nausea in 25 patients (5.8%).

One or more serious AEs occurred in 12 patients (2.8%) and were considered unrelated to the study drug by the investigators. Clinically noteworthy abnormal laboratory values (CNALVs) occurred in 102 of the 430 patients in the safety evaluation. The most common CNALVs were decreased creatinine clearance reported in 63 patients (14.7%), increased aspartate aminotransferase (AST) concentration in 16 patients (3.7%), increased alanine aminotransferase (ALT) concentration in 15 patients (3.5%), increased alkaline phosphatase concentration in 12 patients (2.8%), and increased potassium concentration in 12 patients (2.8%). Most of these CNALVs were present at study entry (61.9%). Furthermore, 55% of all CNALVs did not deteriorate further during the treatment period, or laboratory values returned to within the normal range at the final post-therapy laboratory assessment. One case of decreased creatinine clearance resulting in renal insufficiency noted on Day 1 was considered moderate in intensity by the investigator and not related to the study drug. There were no cases of clinical hepatitis reported during the study.

AEs resulting in discontinuation of telithromycin occurred in 11 patients (2.6%). Six patients who discontinued treatment experienced allergic reaction, abdominal pain, diarrhea, vomiting, vertigo, or increased ALT concentration, which were considered possibly related to telithromycin. The remaining events leading to discontinuation (considered unrelated to telithromycin) were confusion, apnea, lung disorder, respiratory disorder, and myocardial infarction. Two deaths occurred during the study (1 resulting from myocardial infarction and 1 due to renal insufficiency and acute aspiration). Neither death was considered related to telithromycin. There were no clinically significant changes in vital signs from baseline mean or median values. Two patients experienced increases of ≥ 60 ms in QTc intervals, but these were not associated with any AEs and were not considered clinically significant.

## Discussion

Results of this study demonstrate that 7-day treatment with telithromycin 800 mg once-daily telithromycin, the first ketolide antibacterial to undergo clinical development, is safe and effective in the treatment of CAP. Telithromycin was well tolerated and effective in > 90% of clinically and bacteriologically evaluable patients treated according to the study protocol. The favorable results for telithromycin in the present study are consistent with those reported previously in comparative trials. The safety and efficacy of telithromycin 800 mg once daily for varying treatment durations have been compared with those of clarithromycin, amoxicillin, and trovafloxacin. Respective clinical cure rates for 5-day and 7-day telithromycin versus 10-day clarithromycin were 89.3% and 88.8% versus 91.8%; for 10-day telithromycin versus 10-day amoxicillin, 94.6% versus 90.1%; and for 7- to 10-day telithromycin versus 7- to 10-day trovafloxacin, 90.0% versus 94.2% [23-25]. While the optimal treatment duration for CAP has not yet been established, 7-day telithromycin treatment appears to be effective. This shorter, once-daily regimen may encourage patients to complete their prescribed course of medication.

An important consideration in antibacterial treatment of patients with CAP is efficacy in those at increased risk of morbidity. Of particular concern is the possibility of bacteremic infection when it may not be suspected (a situation in which treatment with standard antibiotics could fail). Musher et al [26] have demonstrated that similar proportions of bacteremic cases occur in patients with pneumococcal CAP regardless of disease severity (i.e. among those with PSI scores ranging from I to IV). Telithromycin was highly effective in clinically evaluable patients with bacteremia, including *S pneumoniae *bacteremia. In addition, the clinical outcome in this study in patients ≥ 65 years of age was 95.7% (45/47 cured); in those with PSI scores of III and IV, telithromycin resulted in clinical cure rates of 91.4% and 100%, respectively. In other comparative studies, telithromycin showed comparable or slightly superior efficacy in high-risk patients versus clarithromycin and amoxicillin [23-25].

Telithromycin demonstrated bacteriologic eradication rates of 100% for *S pneumoniae *(57/57 isolates) in the bacteriologically evaluable patient population, including all 9 *S pneumoniae *strains demonstrating resistance to penicillin and/or erythromycin. Telithromycin was also 100% effective against *M catarrhalis *(13/13 isolates eradicated) and 96% effective against *H influenzae *(47/49 isolates eradicated). Susceptibility testing performed in this study revealed that penicillin- and macrolide (erythromycin)-resistant *S pneumoniae *strains showed resistance to clarithromycin, trimethoprim/sulfamethoxazole, and cefuroxime axetil: all 8 strains of penicillin-resistant *S pneumoniae *were resistant to both trimethoprim/sulfamethoxazole and cefuroxime axetil, and 5 (62.5%) were resistant to clarithromycin. Seven of the erythromycin-resistant strains (77.8%) were also resistant to trimethoprim/sulfamethoxazole, and 5 (55.6%) were also resistant to cefuroxime axetil.

The number of atypical/intracellular pathogens isolated from CAP patients in this study was small – in part due to the study design, which did not obtain convalescent serum in patients with initial cultures positive for typical pathogens and excluded patients with cultures positive for typical pathogens from the final analysis of atypical/intracellular pathogens. All cases (3 patients with *C pneumoniae*, 2 with *M pneumoniae*, and 4 with *L pneumophila*) were clinically cured.

The safety profile for telithromycin in this study was similar to that reported in a review of previous clinical trials of this ketolide [7]. Telithromycin was very well tolerated, with the most commonly reported AEs (generally involving the gastrointestinal system [diarrhea and nausea]) being mild to moderate. Study discontinuations occurred in only 6/430 (1.4%) patients due to AEs considered by the investigators to be possibly related to the study drug.

In summary, 7 days of once-daily treatment with telithromycin 800 mg orally was effective and well tolerated in adolescent and adult patients with CAP, including those infected with resistant strains of *S pneumoniae*. Telithromycin may be considered a convenient, well tolerated, and effective treatment option for CAP in this era of increasing antibacterial resistance.

## Competing interests

BPL is an employee of sanofi-aventis and holds stocks in sanofi-aventis. CMF has received research funding for clinical trials from Aventis Pharmaceuticals, a member of the sanofi-aventis Group. All other authors declare that they have no competing interests.

## Authors' contributions

CMF, TCP, and LMD were investigators on the study. BPL participated in the design of the study. All authors read and approved the final manuscript.

## Pre-publication history

The pre-publication history for this paper can be accessed here:


